# Familial Pemphigus Vulgaris Occured in a Father and Son as the First Confirmed Cases

**DOI:** 10.1155/2016/1653507

**Published:** 2016-06-15

**Authors:** Ali Haydar Eskiocak, Birgul Ozkesici, Soner Uzun

**Affiliations:** Department of Dermatology and Venereology, Akdeniz University School of Medicine, 07059 Antalya, Turkey

## Abstract

Pemphigus vulgaris (PV) is a chronic autoimmune bullous disease of the skin and mucous membranes. Although there is some evidence pointing towards a genetic predisposition by some human leukocyte antigen (HLA) genes, familial occurrence of PV is very rare. Most of the familial PV cases so far reported have been in mother and daughter and in siblings. PV in father and son, as presented here, has not been reported in the literature before, except an unconfirmed report. The diagnosis of PV was established by histologic, cytologic studies and enzyme linked immunosorbent assay (ELISA) in Case 1 and by ELISA and BIOCHIP indirect immunofluorescence test in Case 2. The son was responsive to moderate doses of methylprednisolone, with the treatment continuing with tapered doses. The father was in a subclinic condition; consequently, only close follow-up was recommended. HLA typing studies revealed identical HLA alleles of HLA-DR4 (DRB1^⁎^04) and HLA-DQB1^⁎^03 in both of our cases; this had been found to be associated with PV in prior studies. Familial occurrences of PV and related HLA genes indicate the importance of genetic predisposition. The first occurrence of confirmed familial PV in father and son is reported here.

## 1. Introduction

Pemphigus vulgaris (PV) is a life-threatening chronic autoimmune disease, associated with loss of adhesion between keratinocytes and characterised by mucosal and cutaneous blisters and erosions. It is caused by autoantibodies that bind to the keratinocyte surface antigens desmogleins (Dsg) 1 and 3. However, genetic predisposition and exogenous triggering factors play an important role in etiology [1]. Familial pemphigus is very rare and only a few cases have been published. To the best of our knowledge, there are no confirmed familial PV cases in father-son relationships in the literature thus far. Here, we present cases of the son PV patient followed up by our clinic till now and father PV patient diagnosed previously.

## 2. Material and Methods

A 31-year-old man, who was the son of Case 2 described below, was referred to our outpatient clinic in March, 2015, due to having oral lesions for the preceding 8 months. A biopsy, taken from the lesions by another center where the patient had been referred to at the beginning of his complaints, shows intraepidermal dissociation and negative direct immunofluorescence examination. There was no feature in his medical background. Physical examination revealed extensive erosions on the buccal mucosa, soft palate, and oropharynx, with extremely limited epithelized erosions on his chin. The Nikolsky sign was positive and Tzanck smear test showed acantholytic cells in the oral lesions. Anti-Dsg 1 and Anti-Dsg 3 antibodies were found to be positive in titers of 20 and 87 IU/mL in enzyme linked immunosorbent assay (ELISA), respectively (EUROIMMUN, Lübeck, Germany). The BIOCHIP indirect immunofluorescence (IIF) test (EUROIMMUN, Lübeck, Germany) was negative. A diagnosis of mucocutaneous PV was established. Human leukocyte antigen (HLA) typing by polymerase chain reaction-sequence specific primers (PCR-SSP) method showed the following: class 1: A1, A3, B7, B35, C7, and C15; class 2: DRB1^*∗*^04, DRB1^*∗*^11, and DQB1^*∗*^03. Forty-eight mg per day oral methylprednisolone therapy was initiated. The patient achieved remission and the therapy was continued with tapered doses.

A 54-year-old man, the father of Case 1 described above, was referred to our outpatient clinic with recurrent oral lesions after his son had been diagnosed with PV. We learned that his oral erosions began in 1995 and they were followed by cutaneous blisters after a period of 2 months. In accordance with the histopathological examination performed in the medical center that he was referred to first in 1995, he was diagnosed as PV. Oral prednisolone 80 mg/daily was initiated and the dose was reduced after about 2 months. After the treatment had continued for about 5 years with tapered doses, the lesions were completely cleared and he was in remission for about 15 years. New transient lesions have been developing, especially on his palate after consuming tough foods, and then disappearing spontaneously within 3 or 4 days, for the last 2 years. He was operated on for inguinal hernia and recovered from brucellosis 10 and 8 years ago, respectively.

There was neither erosion nor blister, and the Nikolsky sign was negative in dermatological examination at the time he was referred to our clinic. General physical examination was also normal. Anti-Dsg 3 antibodies were positive in titer of 70 IU/mL, while anti-dsg 1 antibody was negative with ELISA. Additionally, BIOCHIP IIF was performed and showed the following results: intercellular intraepidermal staining with IgG in monkey oesophagus substrate and anti-dsg 3 antibody deposits in dsg 3 transfected human embryonic kidney cells were detected. HLA typing by PCR-SSP method showed the following: class 1: A2, A3, B35, C4, and C15; class 2: DRB1^*∗*^04 and DQB1^*∗*^03. The patient's previous diagnosis of PV was confirmed according to the medical history and the positive ELISA and IIF tests. He was informed and close follow-up was recommended, with no requirement of treatment.

## 3. Discussion

Although there is some evidence concerning exogenous triggering factors and genetic predisposition in pemphigus, definite etiology has not been clarified yet [2]. Familial PV is very rare: there are about 45 cases found in the literature so far (Table 1). Two Turkish siblings with simultaneous onset of PV and two Armenian origin siblings with unusual localized variant PV are the only cases reported in Turkey [3, 4]. Most of the familial PV patients reported are in mother and daughter, mother and son, and siblings. To the best of our knowledge, there is no documented familial PV case in father and son except an unconfirmed case reported in 1940 [5]. Immunofluorescence diagnostic techniques were not available at the time of that report; therefore, definite diagnosis of PV could not be established. In this context, our cases hold the distinction of being the first confirmed father and son familial PV. The occurrence of PV in parents and children indicates the important role of inheritance [2]. It is suggested that HLA genes are related to the disease and pemphigus is also thought to be associated with class II alleles such as DR4 and DR6 after serological typing studies [6]. The HLA-DR4 allele is most strongly associated with Jewish PV patients (in almost 90% of cases), especially in Ashkenazi Jews, and 60% of Japanese patients [7, 8]. An increased frequency of HLA-DR4, DQ8, DR6, and DQ5 has also been found within the non-Jewish population [4]. Furthermore, HLA-DQ alleles, such as HLA-DQB1^*∗*^0302 and DQB1^*∗*^0503, are also found to be associated with pemphigus in different ethnic groups with restriction fragment length polymorphism method [8]. Because PV may be associated with some HLA alleles, we investigated the HLA types of our cases. Both of our cases presented identical HLA-DR4 (DRB1^*∗*^04) and HLA-DQB1^*∗*^03 alleles, consistent with the mentioned reports. It has been demonstrated that dsg 3 epitopes, recognized by autoreactive T helper cells, are carried by the aforementioned PV-associated HLA alleles [9]. However, the same antigenic epitopes have been also demonstrated with healthy carrier individuals [9]. Furthermore, the same HLA alleles were demonstrated in both the patients and their healthy first-degree relatives in 2 studies [3]. These findings imply that the existence of the related HLA genes alone is not sufficient for development of pemphigus [2, 8]. The pemphigus autoantibodies were demonstrated in the sera of the first-degree healthy relatives of patients with pemphigus in some previous studies, which can be put forward as evidence of the genetic basis of the disease. An investigative study with 29 patients with pemphigus and 68 first-degree healthy relatives showed the titers of 1/20 and above of circulating antibodies in sera as statistically significant between healthy relatives and healthy controls in rat oesophagus substrates [10]. Another study with the same design similarly also showed higher IIF test positivity in healthy relatives than healthy controls [11]. Circulating anti-dsg antibodies were also demonstrated with ELİSA in %19,4 healthy relatives of pemphigus patients in a study [12]. According to recent findings, related HLA genes and circulating autoantibodies seem not to be sufficient for the disease to develop in first-degree healthy relatives of patients with pemphigus. There may, therefore, be some unknown additional factors for occurrence of clinic manifestations.

There are some points that could be considered as limitations in our observation. The direct immunofluorescence test was negative in Case 1 and could not be performed in Case 2 because of the lack of active pemphigus lesion. However, there was almost no doubt with the diagnosis of PV of the patients, taking into account all of the medical history, clinical, cytological, and histopathological findings, and immunofluorescence studies of the patients. Also, it would be very appropriate to follow up with a study of the HLA types of all first-degree relatives of the cases to demonstrate the HLA allele profile of the family; unfortunately, that was not possible because of the patients' condition.

## 4. Conclusion

First confirmed familial PV cases in father and son are presented here. Although the occurrence of PV in first-degree relatives indicates the importance of genetic predisposition, it is clear that further studies are required to clarify the definite aetiopathogenesis.

## Figures and Tables

**Table 1:** Familial pemphigus vulgaris cases found in the *PubMed* database. PE: pemphigus erythematosus, PF: pemphigus foliaceus, and PV: pemphigus vulgaris.

Author	Date	Disease	Patients
Yamamoto et al. [8]	2011	PV and PF	Daughter with PF and mother with PV; one sister with PF and another sister with PV
Bordel-Gómez et al. [13]	2006	PV	Three brothers/two brothers/a brother and a sister
Kavak et al. [3]	2005	PV	A brother and a sister
Gokdemir et al. [4]	2004	PV	A brother and a sister
Stavropoulos et al. [14]	2001	PV	Two sisters
Starzycki et al. [2]	1998	PV	A mother and a daughter
Revenga-Arranz et al. [15]	1996	PV	Two brothers
Reohr et al. [16]	1992	PV	Two siblings
Feinstein et al. [17]	1991	Pemphigus	Two sisters
Katzenelson Weissman et al. [18]	1990	PV	A mother and a son
Alijotas et al. [19]	1990	PV	Two siblings
Brenner et al. [20]	1990	PV and PE	Two sisters
Laskaris et al. [21]	1989	PV	A sister and a brother/a daughter, a son, and their mother
Graham-Brown and Lister [22]	1987	PV	A mother and a daughter
Spinowitz et al. [23]	1986	PV	Sisters
Brenner et al. [24]	1985	PV	An uncle and a niece
Ahmed and Sofen [25]	1982	PV	A mother and a daughter/a brother and a sister
Sheklakov and Mashkilleison [26]	1965	PV	A mother and a daughter
Miller and Frank [27]	1949	PV	Unknown
